# Working with catatonia: a qualitative exploration of inpatient team emotional responses

**DOI:** 10.1192/bjo.2021.909

**Published:** 2021-06-18

**Authors:** Emma Salter, Linda Pow, Emma Stacey, Victoria Stephens, Paul Beckley, Jenna Oliphant, Lottie Heimes, Courtney Foster

**Affiliations:** 1 Somerset Partnership NHS Foundation Trust; 2 Somerset NHS Foundation Trust

## Abstract

**Aims:**

Child and adolescent mental health (CAMHS) wards treat patients with variable presentations. During diagnosis and treatment, psychiatric professionals use structured criteria, but also honed awareness of countertransference. Unacknowledged emotional responses can produce powerful dynamics and impact patient care.

Limited information exists on possible emotional responses and team dynamics when working with catatonia.

This project aimed to establish common themes relating to staff felt-experience of working with a specific case of catatonia on a Child and Adolescent Mental Health (CAMHS) ward. A secondary aim was to establish potential areas for future training and service improvement.

**Method:**

Trust Research and Development department approved this work. Inpatient professionals working with the specified patient during admission were eligible. Participants were invited via email and face-face discussion with one of the authors. Participants, patient and mother provided written consent.

A questionnaire was created and disseminated via email to eligible staff (n = 33). 27 questions asked individuals to rate responses on Likert scales, plus space for further comments. Questions involved emotional responses to different catatonic states, feelings towards self, patient, colleagues and plans. Descriptive analysis was completed on this anonymised data.

Qualitative data were gathered via 1-hour recorded focus group, led by a systemic psychotherapist and psychologist. The session was transcribed anonymously. Two clinicians, using Thematic Analysis, reviewed the transcript independently.

**Result:**

16 (48.5%) questionnaires were completed. Participants felt negatively about themselves and colleagues more frequently than about the patient. Participants felt positively about themselves less frequently than about colleagues and the patient. Participants identified with more feelings during immobile patient states than lucid states. During immobile states, participants identified with abusive, guilt, hopeless and neglectful responses; during lucid states, with helpful, caring, happy responses

Eight (50%) participants felt they sometimes did not understand their feelings towards colleagues/plans. Nine (57%) participants felt they sometimes did not understand their feelings towards themselves. Ten (66%) participants felt they sometimes did not understand their feelings towards the patient.

Ten (62.5%) participants felt confused by their emotions at least some of the time. Two (12.5%) frequently felt confused by their emotions.

Four participants attended the focus group. Themes included confusion, internal and team conflict.

**Conclusion:**

Working with catatonia involved confusion and team splitting. Staff conflict between plans and morals resulted in painful emotions. Prompt psycho-education within teams working with uncommon presentations was identified as a focus for improvement. The authors plan to explore possible avenues for future teaching, learning and team support.

